# Antilisterial activity of tannin rich preparations isolated from raspberry (*Rubus Idaeus* L.) and strawberry (*Fragaria* X *Ananassa* Duch.) fruit

**DOI:** 10.1038/s41598-025-94731-6

**Published:** 2025-03-25

**Authors:** Michał Sójka, Agnieszka Hejduk, Lidia Piekarska-Radzik, Sylwia Ścieszka, Katarzyna Grzelak-Błaszczyk, Elżbieta Klewicka

**Affiliations:** 1 Institute of Food Technology and Analysis, Lodz University of Technology, Stefanowskiego St. 2/22, Lodz, 90-537 Poland; 2 Institute of Fermentation Technology and Microbiology, Lodz University of Technology, 171/173 Wólczańska St, Lodz, 90-530 Poland

**Keywords:** *Listeria monocytogenes*, Raspberry, Strawberry, Tannins, Antilisterial, Antimicrobials, Natural products, Mass spectrometry

## Abstract

The tannin rich preparations isolated from red raspberry (*Rubus idaeus* L.) and strawberry (*Fragaria* x *ananassa* Duch.) fruits were evaluated for their polyphenol composition and antimicrobial activity against six strains of *Listeria monocytogenes*, sourced from the ATCC collection. The preparations were obtained using solvent extraction with a water-acetone solution, followed by purification using Amberlite XAD 1600 resin. The resulting products, RTRP (raspberry tannin rich preparation) and STRP (strawberry tannin rich preparation), were characterized by their content of ellagitannins, proanthocyanidins, and anthocyanins. Polyphenol content was determined using HPLC-FD and UHPLC-DAD-MS with QExactive mass spectrometer. The antagonistic activity of the preparations against *Listeria* spp. strains was assessed using the disk diffusion method, and minimum inhibitory concentration (MIC) and minimum bactericidal concentration (MBC) values were determined by dilution techniques. The RTRP and STRP exhibited tannin contents of 74 g/100 g and 47 g/100 g, respectively. In the raspberry preparation, ellagitannins were dominant, while in the strawberry preparation, ellagitannins and proanthocyanidins were present at similar levels. In the general antagonism test at a concentration of 60 mg/mL, inhibition zones for *L. monocytogenes* ranged from 10.0 to 24.5 mm. The MIC values for the preparations ranged from 1.563 to 25 mg/mL, varying depending on the tested strains. Based on MIC and MBC, *L. monocytogenes* ATCC 19,111 was the most sensitive to the preparations, whereas ATCC 15,313 exhibited the greatest resistance. Despite their different tannin profiles, the preparations generally did not show statistically significant differences in their antilisterial activity. The results indicate that the tannin rich preparations from red raspberry and strawberry fruits exhibit moderate antilisterial activity, dependent on the sensitivity of the specific *L. monocytogenes* strain tested.

**Supplementary Information:**

The online version contains supplementary material available at 10.1038/s41598-025-94731-6.

## Introduction

According to various scientific studies, tannins have high antimicrobial potential^1^. These compounds, which belong to the polyphenols, can serve as alternatives to food and cosmetic preservatives in line with the “clean label” concept, which promotes replacing synthetic substances with safe and naturally derived preparations^2,3^. Tannins are not an alternative to antibiotics, but they can certainly complement them, especially in therapies against antibiotic-resistant pathogens^4^. The antimicrobial effect of tannins is based on several complex mechanisms. Notably, tannins exhibit the ability to chelate metal ions, inhibit cell wall synthesis, obstruct enzymatic activity, and disrupt fatty acid biosynthesis pathways^1^. Tannins can be divided into two groups based on their structure and chemical reactivity: hydrolysable tannins (such as gallotannins and ellagitannins) and condensed tannins (proanthocyanidins). Besides their antimicrobial properties, these compounds play essential physiological and biochemical roles in various cell types and plant parts, where they accumulate—such as in fruits, leaves, seeds, and roots^5,6^. Tannin compounds isolated from raspberry and strawberry fruits include ellagitannins, with the most abundant being sanguiin H-6, lambertianin C, and agrimoniin^7,8^. In the case of condensed tannins, they primarily consist of procyanidins, exhibiting an average degree of polymerization of 2.1 for raspberries and 5.4 for strawberries^9,10^.

Research conducted by Puupponen-Pimiä et al.^11,12^ indicates that tannins selectively inhibit the growth of pathogenic bacteria, including *Salmonella* spp., *Staphylococcus* spp., and *Escherichia coli*. Additionally, several studies have demonstrated the antimicrobial activity of tannins against *Listeria* spp. Ellagitannins found in pomegranate have been extensively investigated in this context. For example, research by Gullon et al.^13^ reported significant antimicrobial activity of pomegranate extract against *Listeria* spp. - with punicalagin, a monomeric ellagitannin, identified as the primary polyphenolic compound. Other studies have revealed that the ellagitannin-rich fraction obtained from pomegranate had an MIC value of 5 mg/mL against *L. monocytogenes*, and at a dose of 2.5 mg/mL, it significantly reduced the transcription levels of selected virulence genes by over 17 times (*prfA*,* inlA*,* hly*)^14^. High activity against *L. monocytogenes* has also been observed with polyphenol extracts derived from raspberry pomace, which demonstrated an MIC value ranging from 0.39 to 0.59 mg/mL^15^. Conversely, the study by Marić et al.^16^ indicated that polyphenol extracts from raspberry seeds showed limited inhibitory effects on the growth of *L. monocytogenes*. It is important to note that the data available in the literature presents ambiguous findings regarding the antimicrobial activity of tannins, with the MIC values obtained for the tested preparations ranging from 0.1 to 100 mg/mL. Another significant concern is that many studies are based on a single strain of the microorganism. Experiments conducted by Balgacem et al.^17^ suggest that the sensitivity of *L. monocytogenes* to polyphenol preparations depends on the strain, and most studies on the antimicrobial activity of polyphenol preparations are performed for single strains of a given microorganism. Therefore, the aim of this study was to evaluate the antimicrobial activity of tannin rich preparations containing both ellagitannins and proanthocyanidins isolated from raspberry (*Rubus idaeus* L.) and strawberry (*Fragaria* x *ananasa* Duch.), against *L. monocytogenes.* As part of the research, preparations with a high tannin content will be obtained, particularly rich in dimeric sanguiin H-6 and agrimoniin, trimeric lambertianin C, and proanthocyanidins with a relatively low degree of polymerization. The obtained preparations will be fully characterized qualitatively and quantitatively for the main polyphenolic groups using LC-MS techniques with a QExactive Orbitrap mass detector. Preparations containing these compounds, isolated from raspberries and strawberries, have so far been tested on only a few bacterial and fungal strains. The novelty of the research will be microbiological analyses, including studies of antilisterial activity against six strains of *L. monocytogenes* from ATCC collection, selected based on their importance for food safety and microorganism identification. The strains used in the studies come from both humans and animals and include hemolytic and non-hemolytic variants.

## Materials and methods

### Plant material

Preparations rich in tannins were produced following the method described in a previous publication^18^. For this study, deep-frozen red raspberry fruits (5 kg) and strawberries (7.5 kg) were utilised, sourced from Cajdex (Łódź, Poland). The raspberries (*Rubus idaeus* L.) were whole fruits originating from Morocco, while the strawberries (*Fragaria* x *ananassa* Duch.) were whole fruits sourced from Egypt. The fruits were stored in polyethylene bags by the distributor at a temperature of -18 °C before extraction.

#### Tannin extraction

For the extraction of tannins, the fruit pomace remaining after juice extraction was utilised. This stage is essential due to the removal of a significant amount of anthocyanin compounds and a higher concentration of saccharides from the fruits. Previous studies have shown that a substantial portion of tannins is located in insoluble components of the fruits, such as the skin and seeds, and does not transfer to the juice^19^.

Before processing, the fruit was thawed at 4 °C for 16 h and subsequently subjected to technological processing to obtain juice and pomace^18^. The pomace generated from this process constituted 9% of the weight of the processed fruit. This pomace was served as the starting raw material for the extraction of tannins in the next stage. Extraction was carried out using a solution containing acetone, water and formic acid in a volume ratio of 60:40:0.05 (v/v/v). The solvent-to-pomace ratio was 5:1 (v/m). A total of 432 g of raspberry pomace and 672 g of strawberry pomace were used for extraction, which was carried out in three stages in PE containers (extraction cells). In each stage, extraction was performed for 8 h dynamically and 16 h statically. Dynamic extraction involved shaking the cells using an Elmi DOS-10 L orbital shaker (Aizkraukles, Riga, Lithuania) at a speed of 160 rpm. After completing the static extraction, the extracts were filtered through cotton fabric and stored in PE containers at -18 °C. The extraction residue was subjected to further extraction following the same procedure (second and third stages), with the volume of extractant used in each subsequent stage equal to the volume obtained in the preceding stage. The extracts obtained from the three stages were combined to yield 9230 mL of strawberry extract and 5820 mL of raspberry extract. To eliminate any remaining insoluble components of the pomace, the extracts were filtered through a Hobrafilt S40N cellulose filter (Hobra-Školnik S.R.O., Broumov, Czech Republic). In the subsequent step, acetone was removed from the filtered extracts using a laboratory Heidolph rotary evaporator (Schwabach, Germany). After the removal of acetone, 1650 mL of raw raspberry pomace extract and 2400 mL of raw strawberry pomace extract were obtained. The crude tannin extracts were then subjected to a purification process.

#### Tannin purification

The purification of tannins was carried out using low-pressure column chromatography with Amberlite XAD 1600 N (DOW, Midland, USA) sorbent following the methodology described by Klewicka et al.^18^. The purification process used a column measuring 90 cm x 1.6 cm with the sorbent. During the elution of tannin compounds, water-ethanol solutions were employed, with the alcohol concentration increased from 0 to 60%. After the ethanol was removed from the eluates using a laboratory rotary evaporator, the resulting liquid extracts were stored at -60 °C and subjected to freeze-drying under the following conditions: 48 h, 0.2 mbar, -36 °C. In this way, pure, dry preparations rich in tannins, approximately 7 g from raspberries (designated as RTRP - raspberry tannin rich preparation) and approximately 3.5 g from strawberries (designated as STRP - strawberry tannin rich preparation) were obtained. RTRP and STRP were subsequently characterized in terms of their polyphenolic composition and antilisterial activity.

#### Qualitative and quantitative analysis of hydrolysable tannins (ellagitannins)

The identification and quantification of ellagitannins in RTRP and STRP were performed using a UHPLC Ultimate 3000 chromatographic system (Thermo Fisher Scientific, Germering, Germany) equipped with a DAD detector and a QExactive Orbitrap mass detector. Samples for analysis were prepared by dissolving 6 mg of STRP and 3 mg of RTRP in 5 mL of a solution containing 50% methanol and 0.01% formic acid. These solutions were then diluted 1:1 (v/v) with mobile phase A, centrifuged (5 min, 12000 x g), and injected into the chromatographic column. The separation of ellagitannins was carried out using a Luna Omega 1.6 μm C18 100Å column with dimensions of 150 × 2.1 mm (Pehnomenex, Torrance, CA, USA). Phase A consisted of 0.5% (v/v) formic acid in water, while phase B was a mixture of acetonitrile, methanol, water, formic acid in a specific volumetric ratio of (63:20:16.5:0.5 v/v/v/v). The following gradient was applied: 0–2 min, 5% B; 2–12 min, 5–28% B; 12–20 min, 28–73% B; 20–25 min, 73% B; 25–27 min, 73–5% B; 27–35 min, 5% B. The column temperature was maintained at 40 °C, the flow rate was set to 0.4 mL/min, and the injection volume was 5 µL. The parameters for the mass detector were as follows: negative ionization mode, the evaporator temperature was set to 400 °C, electrospray voltage of 4 kV and a spray capillary temperature of 380 °C. Nitrogen drying and auxiliary gas flow rates of 60 and 15 units, respectively. Data were collected in the range of 150–2000 m/z in Full MS and data-dependent MS^2^ modes. Detector optimization was performed by direct injection of RTRP or STRP, which were dissolved in a mixture of mobile phases at a volumetric ratio of phases A and B of 80:20 (v/v), delivered at a flow rate of 0.25 mL/min. UV-Vis detection was carried out in the range of 200–600 nm, with a wavelength of 250 nm selected for quantitative analysis of ellagitannins. The analysis of the identified ellagitannins content was based on standard curves derived from available standards, i.e., sanguiin H-6, lambertianin C, and agrimoniin with HPLC purity (at 210 nm) of approximately 90%, as obtained according to methods described in a previous study^19^. Additionally, a standard curve for ellagic acid purchased from Extrasynthese (Genay, France) was also used in the studies.

#### Analysis of the content of condensed tannins (proanthocyanidins) and free catechins

The content of proanthocyanidins was determined using the method described by Sójka et al.^20^. Briefly, 20 mg of the preparation was weighed into 2 mL plastic test tubes, followed by the addition of 800 µL of a methanol solution containing phloroglucinol (75 g/L) and ascorbic acid (15 g/L), along with 400 µL of 0.4 M HCl dissolved in methanol. The phloroglucinolysis reaction was conducted at 50 °C for 30 min. The sample was then cooled in an ice bath, and 600 µL of 40 mM sodium acetate dissolved in water was added. Prior to chromatographic analysis, the samples were centrifuged at 12,000 x g. Free catechins were determined from solutions prepared by dissolving 5 mg of the sample in 2 mL of a solution containing 50% methanol and 0.01% formic acid, which was further diluted 1:1 (v/v) with mobile phase A and centrifuged at 12,000 x g. The separation of phloroglucinolysis products, including catechin adducts with phloroglucinol, released catechins, and free catechins, was performed using a Shimadzu chromatograph (Tokyo, Japan), equipped with an LC-20AD pump, SIL-20ASHT autosampler, CTO-10ASVP thermostat, and RF-10AXL detector. A Gemini 5u C18 110 A column (Phenomenex, Torrance, USA) with dimensions of 250 mm x 4.6 mm and a 5 μm particle size was used. Mobile phase A consisted of 2.5% acetic acid in water, while mobile phase B was 80% acetonitrile in water. The column temperature was maintained at 30 °C, and the flow rate was 1 mL/min. The following gradient was applied: 0–10 min, 4–7% B; 10–27 min, 7–30% B; 27–29 min, 30–70% B; 29–34 min, 70% B; 34–35 min, 70–4% B; 35–40 min, 4% B. Injection volumes were 10 µL for phloroglucinolysis products and 20 µL for free catechins. Data were collected using LabSolutions software (Shimadzu, Tokyo, Japan). Individual compounds were identified by comparing retention times with standard substances: (-)-epicatechin, (+)-catechin, (-)-epicatechin-phloroglucinol, (+)-catechin-phloroglucinol, and UV-Vis spectra. Quantification was performed for chromatograms (included in the supplementary materials) recorded by an FD detector with an excitation wavelength of 278 nm and an emission wavelength of 360 nm. Calculations were made based on standard curves for the adduct: (-)-epicatechin-phloroglucinol and terminal (-)-epicatechin obtained as a result of the phloroglucinolysis reaction of the procyanidin B2 standard (Extrasynthese, Genay, France). The total proanthocyanidin content was calculated as the sum of the formed adducts and the released terminal units. The average degree of polymerization of proanthocyanidins (mDP) was calculated by dividing the number of moles of all flavan-3-ols (phloroglucinol adducts + terminal catechins) by the number of moles of terminal catechins (sum of released (+)-catechin and (-)-epicatechin). Free catechins were determined on the basis of standard curves determined for (-)-epicatechin and (+)-catechin (Sigma-Aldrich, Steinheim, Germany).

#### Qualitative and quantitative analysis of anthocyanins

The same chromatographic system used for ellagitannin analysis was applied to identify and quantify anthocyanins in RTRP and STRP. Samples were prepared by dissolving 10 mg of the STRP and 15 mg of the RTRP in 2 mL of a solution containing 50% methanol and 0.01% formic acid solution. After diluting 1:1 (v/v) with mobile phase A, the solutions were centrifuged (5 min, 12,000 x g) and injected into the chromatographic column. Anthocyanin separation was performed using a Gemini-XN 3 μm C18 110Å column with dimensions of 150 × 4.6 mm (Pehnomenex, Torrance, CA, USA)3. Phase A was 1% (v/v) formic acid in water, and phase B was 1% (v/v) formic acid in methanol. The following gradient was used: 0–30 min, 20–65% B; 30–31 min, 65–100% B; 31–33 min, 100% B; 33–34 min, 100–20% B; 34–45 min, 20% B. The column was maintained at 35 °C, the flow rate was 0.5 mL/min, and injection volume of 10 µL. The mass detector parameters were as follows: positive ionization mode, vaporizer temperature 400 °C, electrospray voltage 3.8 kV, spray capillary temperature 380 °C; drying and auxiliary nitrogen flow 60 and 15 units, respectively. Data were collected in the range of 250–1000 m/z in FullMS and data dependent MS2 mode. Optimization of the detector operation was performed by direct injection of a solution from the RTRP or STRP diluted in the mixture of mobile phases in a volume ratio phases A and B equal to 80:20 (v/v) and injected at a flow of 0.25 mL/min. The DAD detector operated between 200 and 600 nm, using 520 nm for quantitative analysis. Anthocyanin was determined using standard curve for cyanidin-3-glucoside purchased from Extrasynthese (Genay, France).

#### Microbial material

The antagonistic activity of the RTRP and STRP was tested against six *Listeria monocytogenes* strains from the ATCC collection, marked with the following numbers: 19,115, 19,112, 35,152, 7644, 15,313, and 19,111. These strains were purchased from Microbiologics^®^ (St. Cloud, Minnesota, USA) and cultured on Nutrient Agar (Merck, Germany).

#### Antilisterial activity and minimal inhibitory concentration (MIC)

The determination of the antagonistic activity of tannins against *L*. *monocytogenes* was performed according to the EUCAST guidelines^21^ with modifications as described. To evaluate the antimicrobial properties of RTRP and STRP against *Listeria* spp. biomass from a 24-hour culture was collected using a sterile loop and added to a sterile physiological saline solution to obtain a turbidity corresponding to 1 on the McFarland scale. This suspension was spread onto Muller-Hinton Agar (Merck, Germany) using sterile swabs. Sterile clean paper discs with a diameter of 5 mm (Oxoid Ltd., Basingstoke, UK) were placed on the prepared plates. Then, 20 µL of RTRP and STRP extract solutions prepared earlier by dissolving lyophilized preparations in 5% [v/v] DMSO (Sigma-Aldrich) at a concentration of 60 mg/mL were applied to the discs. After incubating at 37 °C for 18–24 h, the inhibition zones of the test strains were measured in mm. The experiment was performed in triplicate.

To determine the MIC values for the tested preparations, sterile paper discs with a diameter of 5 mm (Oxoid Ltd., Basingstoke, UK) were carefully placed on solidified agar plates. Subsequently, 20 µL of appropriately diluted RTRP and STRP extracts (prepared by dissolving lyophilized preparations in 5% [v/v] dimethyl sulfoxide (DMSO) from Sigma-Aldrich) were applied to the discs. The concentration range of the preparations was 100–0.7815 mg/mL. As a negative control, 5% DMSO was used, while positive controls consisted of discs with the antibiotic chloramphenicol (30 µg/disc, Sigma-Aldrich). The plates were then incubated at 37 °C for 18–24 h. After incubation, the growth inhibition zones of *Listeria* spp. were measured. The MIC represents the lowest concentration within the range of 100–0.7815 mg/mL at which growth inhibition of the bacteria was still observed.

#### Antilisterial activity – minimal bactericidal concentration (MBC)

To determine the MBC values of the tested preparations, cultures of the tested bacterial strains were prepared in Nutrient Broth (Merck, Germany) with the addition of raspberry and strawberry extracts. For this purpose, 200 µL of medium containing the extracts was added to the wells in a 96-well polystyrene plate (the concentration of preparations in the medium was selected based on the MIC value - for each strain, a medium with the addition of preparations at a concentration of MIC and 2MIC was prepared). The wells were then inoculated with a bacterial suspension prepared according to the methodology described above (*MIC* section). The plates were incubated for 24 h at 37 °C. After incubation, bacterial growth was assessed using the drop plate method, as described by Naghili et al.^22^ with slight modifications. The MBC represents the lowest concentration at which a 99.9% reduction in biomass multiplication was observed. The number of bacteria was determined according to formula (1).

#### Determination of bacterial count

The bacterial count was performed using the drop plate method according to Naghili et al.^22^ with modifications. From the prepared tenfold dilutions of the inoculum, 10 µL was taken and surface-seeded onto Nutrient Agar (Merck, Germany) – 5 drops, which corresponds to 50 µL of the culture. After the drops dried, the plates were incubated for 48 h at 37 °C. Following incubation, the number of colony-forming units (CFUs) in each drop was counted, and the average value was calculated. The bacterial count was determined using the following formula:

1 $$CFU/mL{\text{ }} = {\text{ }}\left( {\frac{{Average~CFU}}{{V_{{drop}} \left( {mL} \right)}}) \cdot R} \right)$$

In the case of determining growth or death curves for calculating the bacterial count of *Listeria* spp., the following formula was used:

2 $$Log_{{10}} \left( {CFU/mL} \right){\text{ }} = {\text{ }}Log_{{10}} \left[ {\left( {\frac{{Average~CFU}}{{V_{{drop}} \left( {mL} \right)}}} \right) \cdot R} \right]$$

where:

Average CFU - the average number of colony-forming units in one drop.

V_drop_ - the volume of one drop (0.01 mL).

R - the sample dilution factor.

#### Growth and/or death curves

To determine the growth and/or death curves of *Listeria* bacteria, cultures were prepared with the addition of RTRP and STRP at MIC and 2MIC concentrations. Sterile Nutrient Broth (Merck, Germany) was used, and the preparations were added to achieve the final concentrations corresponding to MIC and 2MIC values for each tested strain. Subsequently, these prepared cultures were inoculated with a bacterial suspension following the methodology described above (*MIC* section). *L. monocytogenes* were introduced into the samples so that the initial density was approximately 8–9 logarithmic units. In this case, the bacterial suspension was standardized on the McFarland scale to a value of 5. The control group consisted of cultures without the addition of preparations. The incubation was conducted at 37 °C for 6 h, and samples were collected at 0, 2, 4, and 6 h using the drop plate method described in the previous point^22^. After 48 h of incubation at 37 °C, the average number of CFUs was counted. The biomass multiplication was calculated according to the formula (3), and the results were presented as ΔLog10 (CFU/mL).

3 $$\Delta Log_{{10}} \left( {CFU/mL} \right){\text{ }} = {\text{ }}Log_{{10}} \left( {CFU/mL} \right)_{T} - {\text{ }}Log_{{10}} \left( {CFU/mL} \right)_{{T0}}$$

where:

T - cultivation time in hours.

T0 - start time of cultivation in hours.

#### Statistics

The results were analyzed using the Statistica 12 software (StatSoft, Tulsa, USA). In the study, one-way ANOVA and Duncan’s post-hoc tests were employed.

## Results

### Characteristics of Ellagitannin preparations - efficiency, appearance

**Fig. 1:**
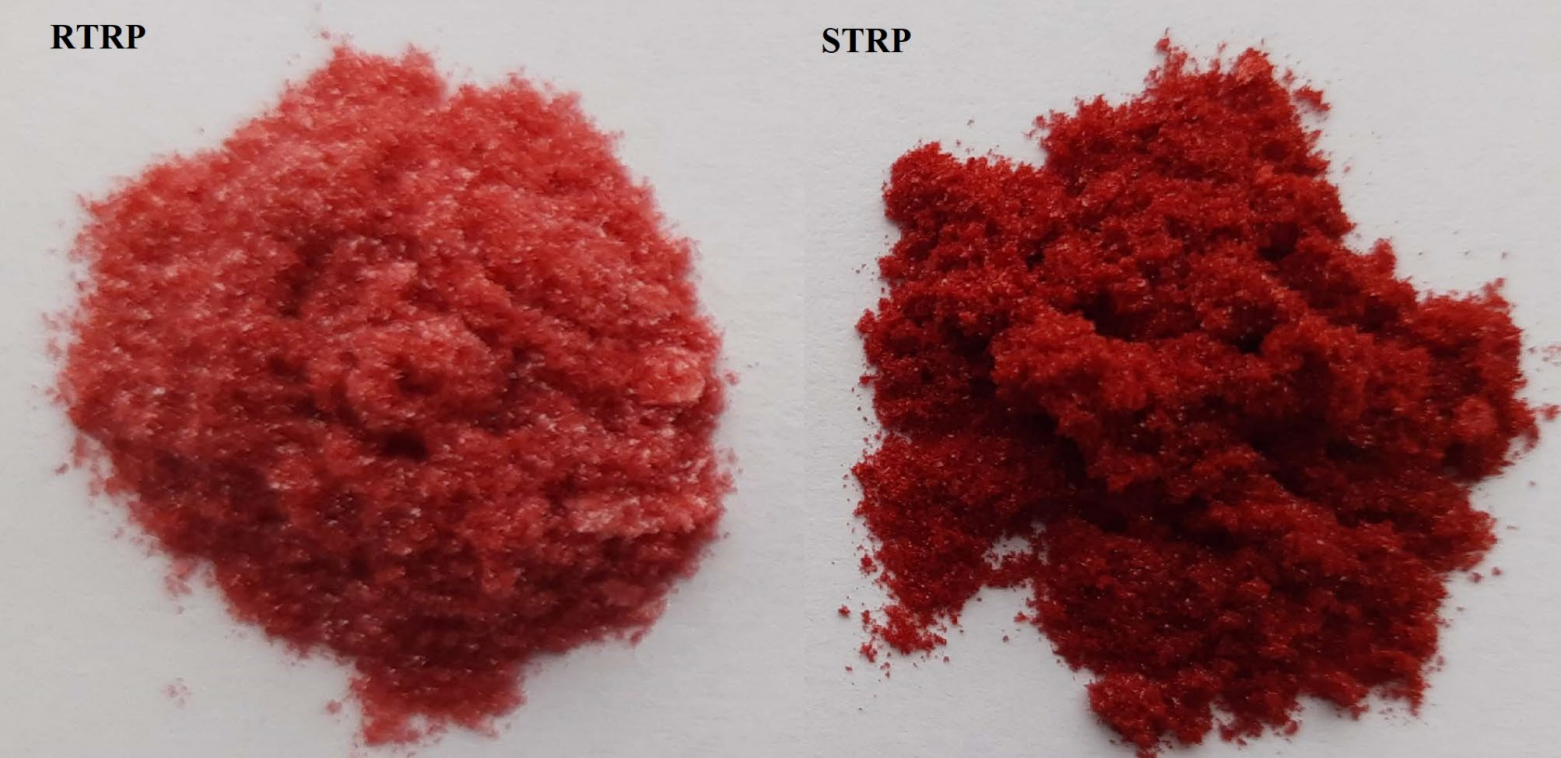
Polyphenolic preparations rich in tannins obtained from raspberries RTRP (left side) and strawberries STRP (right side).

The extraction and purification process of the studied fruits resulted in two preparations rich in tannins. From raspberries, an RTRP was produced in a quantity of 7 g. Considering the amount of raspberries used, the production efficiency of RTRP was 1.4 g/kg of fruit. From strawberries, an STRP was obtained yielding 3.5 g, which corresponds to an efficiency of 0.46 g/kg of fruit. The production efficiencies of RTRP and STRP, calculated based on the fresh pomace mass, were 16.4 g/kg and 5.2 g/kg, respectively. These calculations assumed that the pomace constituted 9% of the mass of fruit intended to produce the mentioned preparations. The juice removed during fruit processing was discarded due to its low ellagitannin content. This indicates that in technological practice, polyphenol preparations can be obtained from pomace, which is waste material after juice production. The purified extracts were preserved through freeze-drying, resulting in powdered preparations with a pink color (RTRP) and a red color (STRP) Fig. 1.

### Polyphenol composition of RTRP and STRP - identification

Table 1 presents the identification of ellagitannin compounds found in the RTRP. The identification process was conducted using available standards, recorded UV-Vis and MS spectra, as well as relevant literature. LC-MS analysis revealed the presence of 12 ellagitannins and ellagic acid. Peaks 1 and 2 corresponded to the compounds with the lowest molecular weight, with pseudomolecular ions observed at m/z 933 and m/z 783, respectively. These compounds were identified as castalagin and pedunculagin/casuariin. The remaining compounds, with the exception of ellagic acid, exhibited masses exceeding 1568 Da, and the signals from the molecular ions recorded in the MS spectra indicated a charge of z = 2. The preparation also contained two isomers of sanguiin H-10 (peaks 4 and 7) and two derivatives of sanguiin H-6 that lacked gallic acid residues (peaks 3 and 6). Peak no. 5 was identified as lambertianin C without the HHDP acid residue; the MS spectrum of this component was characterized by the presence of a doubly charged pseudomolecular ion m/z = 1250. Additionally, the RTRP was also characterized by the presence of sanguiin H-6 (peak 11) and its isomer (peak 8), with the MS spectra of these compounds displaying doubly charged molecular ions at m/z = 934 and exhibiting identical fragmentation patterns. Lambertianin C (peak 10) and lambertianin D (peak 9) were identified as the compounds with the highest molecular weights present in the RTRP, with their pseudomolecular ions yielding signals at m/z 1401 z = 2 and m/z = 1245 z = 3, respectively. Notably, lambertianin D coeluted with a compound that produced a doubly charged molecular ion at m/z = 1018, suggesting the presence of an ellagitannin characteristic of strawberry i.e. fragarin A. The presence of ellagic acid, sanguiin H-6 and lambertianin C was also confirmed based on existing standards.

**Table 1:** Ellagitannin identification in the RTRP obtained from raspberry fruit (*Rubus Idaeus* L.)

Pn	RT [min]	Tentative identification	Detected (m/z)	Z [M-H]^z−^	MS/MS fragment ions m/z	Sum formula	Δppm	Ref.
1	7.07	Castalagin	933.06499	1	631 ([M – H]^−^ – HHDP), 301 (ellagic acid)	C_41_H_26_O_26_	1.7	b, e
2	7.74	Bis-HHDP-glucose (pedunculagin/casuariin)	783.06898	1	481 ([M – H]^−^ – HHDP), 301 (ellagic acid)	C_34_H_24_O_22_	1.1	b, d, e, f
3	9.32	SH-6-galloyl moiety	858.06727	2	1235 ([M – H]^−^ – HHDP-glu); 935 ([M-H]^−^ -bis-HHDP-glu); 783 (bis-HHDP-glu); 633(HHDP-glu-galloyl); 481 (HHDP-glu); 301 (ellagic acid)	C_75_H_50_O_48_	1.8	b, f, i, k
4	9.42	SH-10 isomer	783.06903	2	1235 ([M – H]^−^ – galloyl-glu); 933 ([M – H]^−^ – HHDP-glu-galloyl); 633 (HHDP-glu-galloyl); 331 (galloyl-glucose); 301 (ellagic acid)	C_68_H_48_O_44_	1.2	b, c, d, f, i, k
5	9.98	LC-HHDP moiety	1250.10498	2	1868 ([M – H]^−^ – HHDP-glu-galloyl); 1567 ([M – H]^−^ – bis-HHDP-glu-galloyl) ; 1235 [([M – H]^−^ – galloyl-bis-HHDP-glu-galloyl); 933 (galloyl-bis-HHDP-glu) ; 633 (HHDP-glu-galloyl); 301 (ellagic acid)	C_109_H_74_O_70_	1.0	h, i, k
6	10.18	SH-6-galloyl moiety	858.06748	2	1235 ([M – H]^−^ – HHDP-glu); 935 ([M-H]^−^ -bis-HHDP-glu); 783 (bis-HHDP-glu); 633(HHDP-glu-galloyl); 481 (HHDP-glu); 301 (ellagic acid)	C_75_H_50_O_48_	2.0	b, f, i, k
7	11.70	SH-10 isomer	783.06805	2	1265 ([M – H]^−^ – HHDP); 1103 ([M – H]^−^ – HHDP-glu); 933 ([M – H]^−^ – HHDP-glu-galloyl); 633 (HHDP-glu-galloyl) ; 301 (ellagic acid)	C_68_H_48_O_44_	-0.1	a, b, c, d, f, i, k
8	12.12	SH-6 isomer	934.07237	2	1567 ([M – H]^−^ – HHDP) ; 1235 ([M – H]^−^ – HHDP-glu-galloyl); 935 ([M – H]^−^ – bis-HHDP-glu-galloyl); 633 (HHDP-glu-galloyl); 301 (ellagic acid)	C_82_H_54_O_52_	1.2	a, b, c, d, e, f, i, k
9	12.55	Lambertianin D Fragariin A type	1245.09252 1018.57765	3 2	1867 ([M – H]^−^ – bis-HHDP-glu-galloyl-bis-HHDP-glu-galloyl); 1567 ([M – H]^−^ – bis-HHDP-glu-galloyl-bis-HHDP-glu-galloyl-HHDP; 1235 (bis-HHDP-glu-galloyl-HHDP); 935 (bis-HHDP-glu-galloyl); 633 (HHDP-glu-galloyl); 301 (ellagic acid) 1235; 935 (bis-HHDP-glucose galloyl); 633 (HHDP-glu-galloyl); 301 (ellagic acid)	C_164_H_106_O_104_ C_89_H_58_O_57_	0.1 0.6	e, k i, j
10	12.71	Lambertianin C	1401.10377	2	1869 ([M – H]^−^ – bis-HHDP-glu-galloyl); 1567 ([M – H]^−^ – bis-HHDP-glu-galloyl-HHDP) ; 1235 ([M – H]^−^ – bis-HHDP-glu-galloyl-HHDP-glu-galloyl); 935 ([M – H]^−^ – bis-HHDP-glu-galloyl); 633 (HHDP-glu-galloyl); 301 (ellagic acid)	C_123_H_80_O_78_	-2.2	d, e, f, g, k
11	13.15	Sanguiin H-6	934.07247	2	1567 ([M – H]^−^ – HHDP); 1235 ([M – H]^−^ – HHDP-glu-galloyl) ; 935 ([M – H]^−^ – bis-HHDP-glu-galloyl); 633 (HHDP-glu-galloyl); 301 (ellagic acid)	C_82_H_54_O_52_	1.3	a, b, c, d, e, f, g, k
12	15.05	Ellagic acid	300.99915	1	-	C_14_H_6_O_8_	2.4	c, d, e, f

*Pn* peak number,* RT* retention time,* HHDP* hexahydroxydiphenic acid ,* glu* glucose , a – Kool et al.^50^, b – Lee et al.^27^, c – Kula et al.^51^, d – Kähkönen et al.^29^, e – Hager et al.^52^, f – McDougall et al.^53^, g – Arapitsas et al.^54^, h – Gasperotti et al.^8^, i – Gasperotti et al.^7^, j – Karlińska et al.^55^, k - Chen et al.^56^.

Table 2 presents the identification of ellagitannins characterizing the STRP preparation. Similarly to the RTRP, the STRP contained 12 ellagitannins. The compounds exhibiting the lowest molecular weights were two components, specifically peaks 1 and 3, which were identified as pedunculagin and casuarictin, corresponding to molecular ions at m/z = 783 and m/z = 935, respectively. Similar to the findings related to raspberry, the remaining compounds in the MS spectra were detected as doubly charged ions (z = 2). Both lambertianin C and sanguiin H-6 derivatives were identified within preparation. Peak 4, which generated a molecular ion at m/z = 934 z = 2, was identified as an isomer of sanguiin H-6. The retention time of this compound was notably close to that of the standard differing by only 0.2 min; however, this does not definitively confirm its identity as sanguiin H-6. The molecular ion of peak 5 gave a signal of m/z = 1018 z = 2 and was identified as fragarin A. Peaks number 6, 7, and 9 were identified as derivatives of lambertianin C, with the first two generated signal at m/z = 1250 and z = 2, which was identified as lambertianin C lacking the HHDP acid residue. Compound number 9 was identified as an isomer of lambertianin C – the pseudomolecular ion and fragmentation were similar to those of lambertianin C, but the retention time did not match the standard for this compound. In the case of peak 7, coelution was observed; in addition to the lambertianin C derivative, an additional ion m/z = 1009 z = 2 was detected, which indicates the presence of the sanguiin H-6 derivative with an attached gallic acid residue. A compound of the same mass was also observed in peak 10. The main ellagitannin compound characteristic of the preparation obtained from strawberry fruit was peak 8, identified as agrimoniin (m/z = 934, z = 2); the presence of this component was confirmed by comparing the retention time and spectra with the existing agrimoniin standard. The tested STRP was also characterized by the presence of two compounds that gave a pseudomolecular ion m/z = 933 z = 2 (peaks 2 and 10), which indicates the presence of a dimeric derivative of castalagin - however, this was not confirmed in the literature. The identification of these compounds was challenging because they are characterized by relatively low concentration and poor ionization ability.

**Table 2:** Ellagitannin identification in the STRP obtained from strawberry fruit (*Fragaria X Ananassa* Duch.**)**

Pn	RT [min]	Tentative identification	Detected (m/z),	Z [M-H]^z−^	MS/MS fragment ions m/z	Sum formula	Δppm	Ref.
1	7.83	Bis-HHDP-glucose (pedunculagin)	783.06989	1	481 ([M – H]^−^ – HHDP); 301 (ellagic acid)	C_34_H_24_O_22_	2.3	a, b, c, e, f
2	10.75	Castalagin-dimer type	933.06561	2	1689; 1085; 933; 631; 451; 301 (ellagic acid)	C_82_H_52_O_52_	2.4	-
3	12.58	Bis-HHDP-glucose-galloyl (casuarictin)	935.08032	1	633 ([M – H]^−^ – HHDP); 301 (ellagic acid)	C_41_H_28_O_26_	1.4	a, b, c, f g, i
4	13.02	Sanguiin H-6 isomer	934.07190	2	1567 ([M – H]^−^ – HHDP); 1235([M – H]^−^ – HHDP-glu-galloyl); 935 ([M – H]^−^ – bis-HHDP-glu-galloyl); 633 (HHDP-glu-galloyl); 301 (ellagic acid)	C_82_H_54_O_52_	0.7	a, e
5	13.49	Fragariin A	1018.07624	2	1567; 1059; 935 (bis-HHDP-glu-galloyl); 783 (bis-HHDP-glucose); 633 (HHDP-glu-galloyl); 301 (ellagic acid)	C_89_H_58_O_57_	2.0	d, e
6	13.69	LC - HHDP moiety	1250.60636	2	1869 ([M – H]^−^ – HHDP-glu-galloyl); 1567 ([M – H]^−^ – bis-HHDP-glu-galloyl); 1085 ([M – H]^−^ – bis-HHDP-glu-galloyl-HHDP-glu) 935 (bis-HHDP-glu-galloyl); 783 (bis-HHDP-glu); 633 (HHDP-glu-galloyl); 481 (HHDP-glu); 301 (ellagic acid)	C_109_H_74_O_70_	0.7	a, b, e
7	14.56	LC - HHDP moiety SH-6 + gallic moiety	1250.60693 1009.57284	2 2	1869 ([M – H]^−^ – HHDP-glu-galloyl); 1567 ([M – H]^−^ – bis-HHDP-glu-galloyl); 1085 ([M – H]^−^ – bis-HHDP-glu-galloyl-HHDP-glu) 935 (bis-HHDP-glu-galloyl); 783 (bis-HHDP-glu); 633 (HHDP-glu-galloyl); 481 (HHDP-glu); 301 (ellagic acid) 1085 ([M – H]^−^ – bis-HHDP-glu-galloyl); 935 (bis-HHDP-glu-galloyl); 783 (bis-HHDP-glu); 633 (HHDP-glu-galloyl); 451 (HHDP-galloyl); 301 (ellagic acid)	C_109_H_74_O_70_ C_89_H_56_O_56_	0.0 1.5	a, b, e a, e, g, h
8	14.65	Agrimoniin	934.07299	2	1567 ([M – H]^−^ – HHDP); 1265 ([M – H]^−^ – HHDP, HHDP); 1085 ([M – H]^−^ – bis-HHDP-glu); 935 ([M – H]^−^ – bis-HHDP-glu-galloyl); 783 (bis-HHDP-glu); 633 (HHDP-glu-galloyl); 301 (ellagic acid)	C_82_H_54_O_52_	1.9	a, d, e, f, g
9	15.07	LC type ET	1401.10791	2	1869 ([M – H]^−^ – bis-HHDP-glu-galloyl); 1567 ([M – H]^−^ – bis-HHDP-glu-galloyl-HHDP); 1235 ([M – H]^−^ – bis-HHDP-glu-galloyl-HHDP-glu-galloyl); 1085 (galloyl-bis-HHDP-glu-galloyl); 935 (bis-HHDP-glu-galloyl; 783 (bis-HHDP-glu); 633 (HHDP-glu-galloyl); 481 (HHDP-glu); 301 (ellagic acid)	C_123_H_80_O_78_	0.8	a, b, e
10	15.75	SH-6 + gallic moiety Castalagin dimer type ET	1009.57294 933.06547	2 2	1567 ([M – H]^−^ – HHDP-galloyl); 1235 ([M – H]^−^ – bis-HHDP-glu); 1085 (([M – H]^−^ –bis-HHDP-glu-galloyl); 935 (bis-HHDP-glu-galloyl); 783 (bis-HHDP-glu) 633 (HHDP-glu-galloyl); 451 (HHDP-galloyl); 301 (ellagic acid) 1085 ([M – H]^−^ – bis-HHDP-glu); 933 (castalagin); 783 (bis-HHDP-glu); 631 (castalagin – HHDP) ; 451 (HHDP-galloyl); 301 (ellagic acid)	C_89_H_56_O_56_ C_82_H_52_O_52_	2.6 2.2	a, e, g, h

Pn – peak number; RT – retention time; HHDP – hexahydroxydiphenic acid ; glu – glucose; a – Gasperotti et al.^8^, b – Macierzyński et al.^57^, c - Enomoto^58^, d – Karlińska et al.^55^, e – Kårlund el al^59^, f - Nowicka et al.^60^, g – Abby et al.^61^, h – Duckstein et al.^62^, i - Barbera et al.^37^.

Table 3 presents the identification of anthocyanin compounds responsible for the red color in RTRP and STRP preparations. The identification of anthocyanins was based on UV-Vis spectra, MS data, and literature references. The presence of cyanidin-3-glucoside was also confirmed by comparing with the retention time of the commercial standard.

**Table 3:** Anthocyanins identification in the RTRP and STRP.

Pn.	RT [min]	Tentative identification	Detected (m/z),	Z [M + H]^z+^	MS/MS fragment ions m/z	Sum formula	Δppm	Ref.
REP								
1	8.18	Cyanidin-3-O-sophorodise	611.1611	1	287.0553	C_27_H_31_O_16_	0.7	a, b, c
2	8.76	Cyanidin 3-O-glucosyl-rutinoside	757.2188	1	287.0556	C_33_H_41_O_20_	0.3	a, b, c
3	9.39	Cyanidin-3-O-glucoside	449.1086	1	287.0553	C_21_H_21_O_11_	1.7	a, b, c
4	10.20	Cyanidin-3-O-rutinoside	595.1663	1	287.0553	C_27_H_31_O_15_	0.9	a, b, c
5	10.82	Pelargonidin-3-O-glucoside	433.1137	1	271.0605	C_21_H_21_O_10_	1.8	a, b, c
SEP								
1	9.41	Cyanidin-3-O-glucoside	449.1079	1	287.0552	C_21_H_21_O_11_	0.1	d, e, f
2	10.80	Pelargonidin-3-O-glucoside	433.1131	1	271.0604	C_21_H_21_O_10_	0.4	d, e, f
3	11.71	Pelargonidin-3-O-rutinoside	579.1711	1	271.0603	C_27_H_31_O_14_	0.5	d, e, f
4	13.02	Pelargonidin-3-O-(6’’-malonyl)glucoside	519.1141	1	271.0603	C_24_H_23_O_13_	1.5	d, f
5	13.49	Unknown	771.1053; 501.1036	1	153.0184 339.0505	-	-	-

*Pn* peak number,* RT* retention time, a – McDougal et al.^53^, b – Chen et al.^63^, c – Mullen et al.^64^, d – Nowicka et al.^60^, e – Mustafa et al.^65^, f – Abby et al.^61^.

In RTRP, cyanidin glycosides such as sophoroside, glucosyl-rutinoside, glucoside, and rutinoside, as well as pelargonidin glucoside, were identified. In STRP, pelargonidin glycosides such as glucoside, rutinoside, malonyl-glucoside, and cyanidin glucoside were present. This preparation also showed the presence of one anthocyanin that was not identified due to an insufficient analytical signal.

### Quantitative polyphenol composition of RTRP and STRP

Tables 4 and 5 provide a quantitative composition of polyphenolic compounds in the RTRP and STRP. Both preparations were characterized by a high content of tannins (ellagitannins + flavanols), with respective contents of 71,646 mg/100 g for RTRP and 44,441 mg/100 g for STRP. In the RTRP, ellagitannins accounted for 88% of the total determined polyphenols, while flavanols comprised 12%. A slightly different share of these two groups of tannins was observed in the STRP, where ellagitannins constituted 43% and flavanols 53% of determined polyphenols. Notably, in both preparations, tannins constituted approximately 95% of the total polyphenols.

**Table 4:** Polyphenol content [mg/100 g] in the RTRP.

Pn	RT [min]	Compound	Mean *n* = 3 [mg/100 g]	SD [mg/100 g]	RSD [%]
Ellagitannins					
1	7.07	Castalagin	247.3	20.5	8.3
2	7.74	Bis-HHDP-glucose (pedunculagin/casuariin)	249.5	18.6	7.4
3	9.32	SH-6-galloyl moiety	226.8	2.8	1.2
4	9.42	SH-10 isomer	523.1	16.4	3.1
5	9.98	LC-HHDP moiety	832.8	22.2	2.7
6	10.18	SH-6-galloyl moiety	500.1	20.8	4.2
7	11.70	SH-10 isomer	787.5	13.3	1.7
8	12.12	SH-6 isomer	980.8	17.0	1.7
9	12.55	Lambertianin D + fragarin A type	2640.9	130.4	4.9
10	12.71	Lambertianin C	35302.4	423.8	1.2
11	13.15	Sanguiin H-6	23130.4	321.3	1.4
12	15.05	Ellagic acid	167.0	5.3	3.2
-	-	Total ellagitannins	65361.8	789.7	1.2
Anthocyanins					
1	8.18	Cyanidin-3-O-sophorodise	114.9	1.1	1.0
2	8.76	Cyanidin 3-O-glucosyl-rutinoside	34.8	1.6	4.6
3	9.39	Cyanidin-3-O-glucoside	86.3	2.1	2.4
4	10.20	Cyanidin-3-O-rutinoside	25.5	1.3	5.2
5	10.82	Pelargonidin-3-O-glucoside	6.0	0.4	5.9
-	-	Total anthocyanins	267.5	6.0	2.2
Flavanols					
		(+)-catechin	195.1	0.9	0.5
		(-)-epicatechin	2471.1	72.9	3.0
-	-	Proanthocyanidins	6284.5	110.6	1.8
		% extCat	36.4	0.2	0.6
		% extEpi	1.9	0.0	1.5
		%tCat	11.2	0.1	0.7
		%tEpi	50.6	0.3	0.6
		mDP	1.62	0.01	0.4
-	-	Total flavanols	8957.9	164.5	1.8

*Pn* peak number, Values are means ± standard deviation (SD),* RSD * relative standard deviation, *n* = 4, %extCat – percent of extender (+)-catechin, %extEpi – percent of extender (-)-epicatechin, %tCat – percent of terminal (+)-catechin, %tEpi – percent of terminal (-)-epicatechin, mDP – mean degree of polymerisation.

**Table 5:** Polyphenol content [mg/100 g] in the STRP.

Pn	RT [min]	Compound	Mean *n* = 3 [mg/100 g]	SD [mg/100 g]	RSD [%]
Ellagitannins					
1	7.83	Bis-HHDP-glucose (pedunculagin)	145.8	5.8	4.0
2	10.75	Castalagin-dimer type	161.0	1.3	0.8
3	12.58	Bis-HHDP-glucose-galloyl (casuarictin)	1081.5	70.5	6.5
4	13.02	Sanguiin H-6 isomer	1589.9	90.8	5.7
5	13.49	Fragariin A	2217.9	81.8	3.7
6	13.69	LC-HHDP moiety	2257.4	116.1	5.1
7	14.56	LC-HHDP moiety, SH-6 + gallic moiety	553.5	40.2	7.3
8	14.65	Agrimoniin	10248.0	49.9	0.5
9	15.07	LC type ET	1854.7	29.8	1.6
10	15.75	SH-6 + gallic moiety, Castalagin dimer type ET	408.9	21.5	5.2
		Total ellagitannins	20211.9	480.2	2.4
Anthocyanins					
1	9.41	Cyanidin-3-O-glucoside	49.0	2.1	4.3
2	10.80	Pelargonidin-3-O-glucoside	1567.5	8.4	0.5
3	11.71	Pelargonidin-3-O-rutinoside	145.9	1.4	1.0
4	13.02	Pelargonidin-3-O-(6’’-malonyl)glucoside	15.1	0.1	0.5
5	13.49	Unknown	3.6	0.1	2.0
		Total anthocyanins	1781.1	11.9	0.7
Flavanols					
		(+)-catechin	711.5	8.7	1.2
		(-)-epicatechin	**-**	**-**	**-**
		Proanthocyanidins	24229.9	176.4	0.7
		% extCat	22.8	0.1	0.4
		% extEpi	56.8	0.1	0.1
		%tCat	19.9	0.1	0.7
		%tEpi	0.5	0.0	5.6
		mDP	4.91	0.05	1.0
		Total flavanols	24943.8	168.4	0.7

*Pn* peak number, Values are means ± standard deviation (SD); *RSD * relative standard deviation; *n* = 4, %extCat – percent of extender (+)-catechin, %extEpi – percent of extender (-)-epicatechin, %tCat – percent of terminal (+)-catechin, %tEpi – percent of terminal (-)-epicatechin, mDP – mean degree of polymerisation.

The total content of ellagitannins in the RTRP was measured at 65,361 mg/100 g, with the predominant compounds identified as lambertianin C (peak 10) and sanguiin H-6 (peak 11). Collectively, these two compounds accounted for nearly 90% of the total ellagitannins quantified. Additionally, peak no. 9, i.e. lambertianin D in conjunction with a fragarin derivative, was present at a concentration of 2640 mg/100 g, representing 4.2% of the total ellagitannins. The remaining compounds were detected at concentrations below 1000 mg/100 g. The ellagic acid content in this preparation was recorded at 167 mg/100 g. Furthermore, RTRP was characterized by anthocyanin content of 267 mg/100 g, where the dominant compounds were cyanidin-3-sophoroside (peak 1) and cyanidin-3-glucoside (peak 3), which together comprised over 75% of the anthocyanins determined. Other anthocyanins were present in amounts less than 35 mg/100 g. The flavan-3-ol content in RTRP was at the level of 8958 mg/100 g, of which 70% were polymeric compounds, i.e. procyanidins. Among the free catechins, the dominant compound was (-)-epicatechin, the content of which was almost 2500 mg/100 g. Procyanidins present in this preparation were characterized by a low degree of polymerization of 1.62, and the percentage of catechins in their structure was as follows: 52.5% (-)-epicatechin, 47.5% (+)-catechin.

The strawberry fruit preparation STRP, exhibited an ellagitannin content that was more than three times lower than that of the preparation derived from raspberries. The total content of these compounds was measured at 20,212 mg/100 g, with agrimoniin (peak 8) being the predominant ellagitannin, accounting for over 50% of the determined ellagitannins. Relatively large amounts of lambertianin C without the ellagic acid residue (peak 6), fragarin A (peak 5), lambertianin C isomer (peak 9) and sanguiin H-6 isomer (peak 4) were also present, these compounds constituted 11.1%; 10.9%; 9.2% and 7.8% of the determined ellagitannins, respectively. The content of anthocyanins in the STRP was more than six times higher than in the RTRP and amounted to 1781 mg/100 g. The dominant anthocyanin in the preparation obtained from strawberry fruit was pelargonidin-3-glucoside, the content of which was at the level of 1567 mg/100 g, which constituted 88% of the anthocyanins determined. The remaining compounds occurred in amounts below 146 mg/100 g. The content of flavan-3-ols in the STRP was at the level of 24,944 g/100 g, which was almost three times higher than in the RTRP. Polymeric compounds (procyanidins) constituted 97% of all the flavanols determined. The presence of free (-)-epicatechin was not demonstrated in the STRP, and the content of (+)-catechin was at the level of 0.7 g/100 g. The determined procyanidins were characterized by a degree of polymerization close to 5, and the percentage of catechins in their structure was as follows: 57.3% (-)-epicatechin, 42.7% (+)-catechin.

### Antimicrobial activity of RTRP and STRP

Table 6 presents the antimicrobial activity of the obtained preparations against six strains of *L. monocytogenes* from the ATCC collection. In the test of the general antagonistic activity of the RTRP and STRP at a concentration of 60 mg/mL using the disk diffusion method, the ability to limit the growth of all tested strains was observed. The zones of inhibition ranged from 10.0 mm to 24.5 mm. The strongest inhibitory effect was noted for *L. monocytogenes* ATCC 19,111, with growth inhibition diameters of 20.0 ± 4.24 mm for the RTRP extract and 24.5 ± 6.36 mm for the STRP extract. For *L. monocytogenes* ATCC 19,112, 35,152, 7644, and 15,313, the inhibition zones ranged from 10.5 to 11.5 mm, regardless of the preparation. The control chloramphenicol at a dose of 30 µg/disc was characterized by a zone of inhibition above 30 mm. In the general antagonism test, RTRP and STRP did not show statistically significant differences in their antilisterial activity despite the different polyphenol composition. However, the variable sensitivity of the tested strains to the effects of the preparations was confirmed.

**Table 6:** General antagonism of the RTRP and STRP (60 mg/mL).

Strain	Growth inhibition zone [mm]				
	RTRP	STRP	Chloramphenicol 30 µg/disc	DMSO 0.05 g/mL	
*L. monocytogenes* ATCC 19,115	16.0 ± 5.66ABa	11.5 ± 0.71Aa	31.0 ± 1.41	0.0 ± 0.00	
*L. monocytogenes* ATCC 19,112	11.5 ± 0.71Aa	11.5 ± 0.71Aa	30.0 ± 0.00	0.0 ± 0.00	
*L. monocytogenes* ATCC 35,152	11.0 ± 0.00Aa	11.0 ± 0.00Aa	31.0 ± 1.41	0.0 ± 0.00	
*L. monocytogenes* ATCC 7644	10.5 ± 0.71Aa	11.0 ± 1.41Aa	31.0 ± 1.41	0.0 ± 0.00	
*L. monocytogenes* ATCC 15,313	11.0 ± 0.00Aa	10.0 ± 0.00Aa	33.5 ± 2.12	0.0 ± 0.00	
*L. monocytogenes* ATCC 19,111	20.0 ± 4.24Ba	24.5 ± 6.36Ba	31.0 ± 1.41	0.0 ± 0.00	

a – effect of extracts on the growth inhibition zone of a specific one microorganism (rows), A, B – effect of one extract on a given type of microorganism (column), Duncan’s post-hoc test (*p* ≤ 0.05).

The determined MIC and MBC values ​​for the tested strains were within the range of 1.563–25 mg/mL and 3.12–100 mg/mL, respectively (Table 7). Comparison of the MIC and MBC values showed that *L. monocytogenes* ATCC 19,111 and ATCC 35,152 were the most sensitive strains. Their MIC and MBC values ​​were identical (1.563 and 3.12 mg/mL, respectively), regardless of the preparation. The most resistant strain to RTRP and STRP was *L. monocytogenes* ATCC 15,313, for which the MIC and MBC values ​​were 16-fold higher. These data, similarly to the general antagonism test, confirm the variable strain sensitivity to the tested preparations. Additionally, for the *L. monocytogenes* ATCC 19,115, ATCC 19,112, and ATCC 7644, differences were observed between the preparations. For *L. monocytogenes* ATCC 19,115 and 19,112, the STRP was characterized by higher MIC and MBC values, compared to RTRP, which suggests a lower inhibitory and biocidal capacity of the polyphenolic preparation obtained from strawberry fruit. On the other hand, an inverse relationship was observed for the *L. monocytogenes* ATCC 7644. Considering the above, it can be concluded that the sensitivity of the tested strains depends on the polyphenolic composition, where STRP containing a large share of condensed tannins strongly inhibit the development of the *L. monocytogenes* ATCC 7644, while RTRP rich in ellagitannins strongly inhibit the development of *L. monocytogenes* ATCC 19,115 and 19,112.

**Table 7:** Minimum inhibitory concentration (MIC) and minimum bactericidal concentration (MBC) of the RTRP and STRP [mg/mL].

Strain	RTRP		STRP	
	MIC mg/mL	MBC mg/mL	MIC mg/mL	MBC mg/mL
*L. monocytogenes* ATCC 19,115	6.25	12.5	25	50
*L. monocytogenes* ATCC 19,112	6.25	12.5	6.25	100
*L. monocytogenes* ATCC 35,152	1.563	3.12	1.563	3.12
*L. monocytogenes* ATCC 7644	3.125	6.25	1.563	3.12
*L. monocytogenes* ATCC 15,313	25	50	25	50
*L. monocytogenes* ATCC 19,111	1.563	3.12	1.563	3.12

### Growth or death dynamics of Listeria monocytogenes

**Fig. 2:**
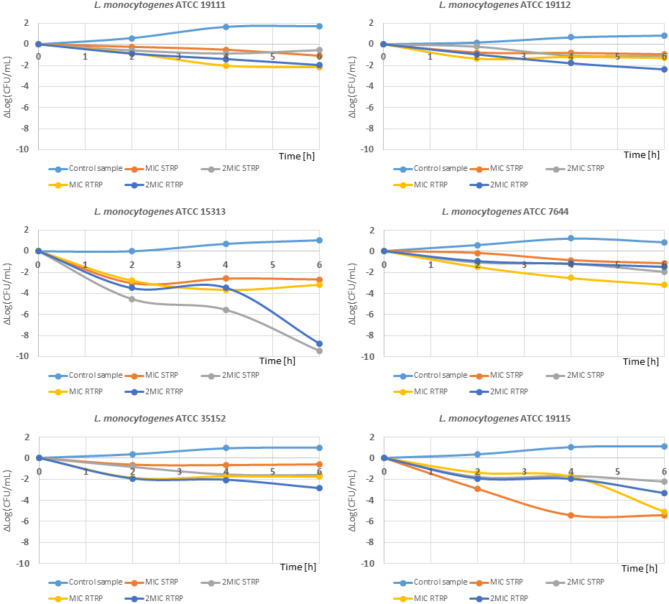
Changes in the number of *Listeria monocytogenes* bacteria in the presence of RTRP and STRP during 6-hour incubation. A positive value indicates an increase in cells above the initial level, while a negative value indicates a decrease below it.

Figure 2 shows the growth dynamics of *L. monocytogenes* in the presence of RTRP and STRP. In the control samples for all six strains of *L. monocytogenes*, bacterial growth was observed during incubation. After 6 h of incubation, the bacterial count increased by 0.85 to 1.11 logarithmic units, yielding an average of 0.986 ± 0.1108 logarithmic units under control conditions. RTRP and STRP, at MIC and 2×MIC concentrations (which corresponds to MBC for five strains) limited the growth of the tested strains of *L. monocytogenes*. The most sensitive strain to RTRP and STRP was *L. monocytogenes* ATCC 15,313 (Fig. 2), where after hours of incubation, a reduction in the number of bacteria was achieved by 9.46 logarithmic units for STRP at a concentration of 2×MIC and by 8.37 logarithmic units for RTRP also at a concentration of 2MIC. In this case, after 6 h of exposure to RTRP and STRP at a concentration of 2MIC, no living cells of *L. monocytogenes* ATCC 15,313 were detected in the tested system. An equally sensitive strain was *L. monocytogenes* ATCC 19,115, for which after 6 h of incubation, a reduction in the number of bacteria was observed by more than 5 logarithmic units for the MIC concentration for both the RTRP and STRP. Surprisingly, for the 2×MIC concentration, the population of this strain decreased after 6 h only by 2 and 2.5 logarithmic units for STRP and RTRP, respectively. The remaining four *L. monocytogenes* strains also responded with a decrease in population count to the presence of RTRP and STRP in the growth medium at the tested time and concentrations. For these strains, an average decrease in bacterial numbers of 2 logarithmic units was observed.

## Discussion

In the studies presented, the antimicrobial properties of tannin rich preparations, specifically those rich in ellagitannins and flavan-3-ols derived from raspberry and strawberry fruits, were evaluated against strains of *L. monocytogenes*.

The procedure employed to isolate the aforementioned compounds has been used in previous studies^18^. The preparations were produced using pomace obtained after pressing juice from the pulp. According to research conducted by Milczarek et al.^23^, pomace, particularly that from juice production, retains 50–70% of the ellagitannins originally present in the fruit. Due to its lower sugar and organic acid content, coupled with a simultaneous higher concentration of polyphenolic compounds compared to whole fruits, pomace represents a promising raw material for obtaining polyphenols, in particular tannin compounds^20,24^. The isolation process involved three stages of extraction using acetone, which effectively isolated both ellagitannins and flavan-3-ols. Research conducted by Milczarek et al.^25^ clearly confirms that a two-stage acetone extraction method allows for the extraction of both low- and high-molecular-weight ellagitannins from raspberry pomace. It should be emphasized that the use of water-acetone solutions in the process of extracting ellagitannins and proanthocyanidins, especially in combination with various supporting techniques, e.g. UAE – Ultrasound Assisted Extraction, MAE – Microwave assisted extraction, ASE – accelerated solvent extraction, is commonly used methods for extracting by-products like pomace^26–28^. The application of Amberlite XAD 1600 in the purification process allowed to obtain extracts with a high content of ellagitannins (> 65%) in the case of the raspberry RTRP, which is comparable to the results obtained by Kähkönen et al.^29^, who, using a similar extraction technique (acetone) and purification method (Amberlite XAD-7), produced an extract characterized by the content of ellagitannins of 60 g/100 g and anthocyanins at 8 g/100 g. In the case of the strawberry STRP, the tannin content was at the level of 45 g/100 g, with ellagitannins and condensed tannins present in similar level. In the study by Fotschki et al.^30^, the authors obtained a polyphenolic preparation from strawberries characterized by a polyphenol content of 79 g/100 g, with ellagitannins and procyanidins comprising 59 and 17% of the determined polyphenols, respectively. Additionally, Kähkönen et al.^29^ indicate that ellagitannins can be separated from anthocyanins and flavonols in a polyphenolic preparation obtained from the fruits of the *Rubus* genus (*Rubus idaeus* L. and *Rubus chamaemorus* L.), However, this process requires an additional purification step using a column filled with Sephadex LH-20.

All identified ellagitannins in the RTRP were previously observed in raspberry fruit. A comparable situation occurs with the STRP – with the exception of two castalagin derivatives (peaks 2 and 10), for which the signal was too weak to allow for full identification using the MS technique. When analyzing the compounds studied, it is worth noting that the presence of identified ellagitannins in both the RTRP and STRP may reflect the specific fruit varieties used^7,8^ as well as the effects of the extraction and purification processes employed. The analysis of anthocyanin compounds also showed that the identified pigments in the RTRP and STRP are characteristic of the fruits from which they were obtained. According to literature data, flavan-3-ols present in raspberry and strawberry fruits are dimers and oligomers of catechin and epicatechin, and in the case of strawberries additionally afzelchin and epiafzelchin^9,10,31,32^. The average degree of polymerization of proanthocyanidins, as reported by Gu et al.^10^, is 2.1 for raspberries and 5.4 for strawberries. The proanthocyanidin profile and degree of polymerization of the obtained RTRP and STRP were similar to the data obtained by Gu et al.^10^. In the case of RTRP, catechin was the dominant extension unit (36%), while epicatechin was the predominant terminal unit (51%). In STRP, epicatechin was the dominant extension unit (57%), whereas catechin was the leading terminal unit, accounting for 20%.

According to Farha et al.^1^, the antibacterial mechanism of tannins should be considered at three levels. The first level involves the antibacterial effects resulting from various factors, including the impact on cell wall membranes, inhibition of cell wall synthesis, and chelation minerals. The antibacterial mechanism of tannins may be the result of its direct binding to peptidoglycan in the bacterial cell wall, interfering with integrity^33^. Another mechanism of action of tannins and their derivatives may be the disruption of fatty acid synthesis in bacteria. In the studies of Wu et al.^34^ it was shown that tannic acid inhibited b-ketoacyl-ACP reductase (FabG), which is an important enzyme in bacterial fatty acid synthesis. It is known that fatty acids, mainly phospholipids, are components of the external structures of the bacterial cell - the cytoplasmic membrane and the cell wall. Disruption of the synthesis of this component carries serious consequences for the cell in the form of reorganization of the structure of these structures, which is associated with its basic protective and transport functions.

The second level pertains to the effects on virulence factors, which include the inhibition of enzymes, prevention of biofilm formation, and blockage of communication channels (*quorum sensing - QS*). The above statements are confirmed by the research conducted by Oliveira et al.^35^ where it was shown that extracts obtained from *Rubus rosaefolius* with a total phenolic content of 5902.89 mg GAE/L effectively limited bacterial intercellular communication (QS) by blocking violacein production, swarming motility and biofilm formation.

The last level addresses the reduction of multidrug resistance by blocking efflux pumps, inhibiting the activity of β-lactamase and inhibiting antibiotic binding proteins. According to the studies by Puupponen-Pimiä et al.^11,12^, tannins selectively inhibit the growth of bacteria pathogenic to humans such as *Salmonella* spp., *Staphylococcus* spp., *E. coli*. Extracts at a concentration of 1 mg/mL obtained from raspberries and strawberries showed particularly high activity against *E. coli* CM871. In the agar diffusion method, lyophilized fruit extracts in the amount of 0.8–7 mg per well showed an *E. coli* inhibition zone of 14–21 mm. In the studies by Ispiryan et al.^36^, polyphenolic extracts obtained from raspberry fruits, as well as from seeds, leaves, roots, stems by ethanol extraction, showed a similar inhibition capacity against *L. monocytogenes*. Inhibition zones in the disk diffusion test were in the range of 16–21 mm. In turn, in the studies conducted by Khalifa et al.^37^ commercial raspberry and strawberry extracts dissolved in water showed activity against *L. monocytogenes* (4b, F2395), where the MIC and MBC values ​​were 2.5 and 10 mg/mL for the raspberry extract and 5 and 10 mg/mL for the strawberry extract, respectively. These researchers also observed the influence of the extract pH on the inhibitory and killing capacity, extracts neutralized to pH 7 significantly reduced their activity against the tested bacteria, and the MIC and MBC values, regardless of the extract and the tested strains, were above 100 mg/mL. In the studies conducted by Marić et al.^16^, polyphenolic extracts obtained from defatted raspberry seeds showed negligible activity against *L. monocytogenes*. In these studies, the inhibition zone against the *L. monocytogenes* ATCC 19,111, using 6 mm discs and 10 µL of extract, was between 7 and 9 mm, which suggests a lack of antimicrobial properties. In our studies, using 5 mm discs and 20 µL of extract, the inhibition zone against the same ATCC 19,111 strain was at the level of 20 mm for the raspberry preparation and 24.5 mm for the strawberry preparation. Hence, it should be emphasized that according to Marić et al.^16^, the extracts used for microbiological tests were obtained by extraction with ethanol solution. These extracts were not subjected to further purification processes, and the content of total ellagic acid (released from ellagitannins) was estimated at a level close to 1 g/100 g, which suggests a relatively low content of polyphenols in this extract. These authors did not estimate the total content of polyphenolic compounds. In turn, studies conducted by Četojević et al.^15^ show that extracts obtained from raspberry pomace from the ‘Meeker’ and ‘Willamette’ varieties are characterized by high activity against *L. monocytogenes* for the strain, which was marked by the authors as wild strain. In these studies, extracts containing 2.6 and 4.4 g/100 g of polyphenols showed activity expressed as MIC and MBC at the level of 0.4–0.6 mg/mL and 0.8 mg/L, respectively. Comparing these results to our results, the values ​​are two to several times lower, which suggests very good activity of these extracts against this strain.

Considering the dynamics of bacterial growth (Fig. 2) in the presence of RTRP and STRP, the number of bacteria decreased during 6 h of incubation. These data clearly indicate that the tested preparations, at the applied MIC and 2×MIC doses have antilisterial activity. However, the findings presented by Barbieri et al.^38^ indicate that the polyphenol preparation obtained from blackberry leaves (*Rubus fruticosus*), at a dose of 0.5×MIC, initially acted in a bacteriostatic manner, as no increase or decrease in bacterial count was observed during the first hours of incubation. After 15 h of incubation, however, an increase in bacterial count was noted. According to these authors, the use of plant extracts has an inhibitory effect by extending the adaptation phase, resulting in a final bacterial count (after 150 h of incubation) that is lower by 1 log unit compared to the control sample. In the studies of Balgacem et al.^17^, the addition of pomegranate peel extract, which is rich in ellagitannins, at doses ranging from 1.2 to 12 mg/mL caused the CFU content of selected *L. monocytogenes* strains to drop below the detection limit, although it should be mentioned that in these studies the maximum interaction time of the preparation with bacteria was 30 min. The authors of these studies also recognize the potential application of such polyphenol preparations in reducing pathogenic microorganisms, particularly in fresh-cut fruit.

The literature indicates that the antimicrobial properties of fruit preparations are associated with the presence of tannin compounds, including both hydrolysable tannins and proanthocyanidins, which exhibit a slightly different mechanisms of action. It is widely accepted that the antimicrobial activity of polyphenols results from the presence of phenolic hydroxyls, which can influence various factors responsible for the viability of microorganisms^1^. In the case of ellagitannins, antimicrobial activity may be related to the presence of galloyl and valoneoyl groups. Research conducted by Shimozu et al.^39^ on *Staphylococcus aureus* and *Enterococcus faecium* revealed that ellagitannins containing only HHDP groups, without free galloyl groups, did not exhibit antimicrobial properties. Conversely, the research by Li et al.^40^ showed that an extract obtained from pomegranate, primarily composed of punicalagin devoid of galloyl groups, caused plasmolysis and damage of cell membranes of *L. monocytogenes* CMCC54004. Both RTRP and STRP extracts obtained in our study contained ellagitannins, which have both HHDP and galloyl groups in their structure, with the main ellagitannins being dimers or trimers of castalagin/potentillin. According to the data presented by Funatogawa et al.^41^, oligomers are characterized by lower antimicrobial activity against *H. pylori* than monomers. The antimicrobial activity of ellagitannins may also be the effect of the release of ellagic acid. Savic et al.^42^ reported that ellagic acid itself shows activity against *L. monocytogenes*. In their studies, the inhibition zone against the ATCC 19,166 strain, using the disk diffusion method, was at the level of 20–22 mm, with a disk diameter of 12.5 mm and a solution of ellagic acid with a concentration of 1 mg/mL in the amount of 60 µL was used. In our studies, the RTRP was characterized by the presence of ellagic acid at the level of 167 mg/100 g; however, it remained undetermined whether the presence of this compound influences the antimicrobial activity. Therefore, further research is necessary to ascertain whether *L. monocytogenes* has an enzymatic apparatus, including tannase, capable of degrading ellagitannins^43,44^ and whether an increase in the concentration of this compound in the preparation can translate into antilisterial activity. The studies by Rappin et al.^45^ indicated that extracts from fruits, including raspberries and strawberries, containing tannins subjected to enzymatic hydrolysis using tannin acyl hydrolase increased both their antioxidant and antimicrobial activity.

Condensed tannins also exhibit antimicrobial activity against *L. monocytogenes*^46,47^. The activity of these compounds is related to both the type of monomers^48^ and the degree of polymerization^1^. In the studies by Sivakumaran et al.^49^, a significant effect of the activity of condensed tannins on rumen bacteria was confirmed, with an increase in the degree of polymerization typically associated with a decrease in its activity. In the research by Wang et al.^47^, procyanidin A1 showed antimicrobial activity against *L. monocytogenes* ATCC 7644, with MIC values ​​for this compound at 64 µg/mL, other procyanidins such as procyanidin B3, procyanidin C4 had MIC values ​​>128 µg/mL, which the authors considered as a lack of antimicrobial activity. In our study, the activity of the STRP, rich in procyanidins, against the same strain, expressed as MIC was at the level of 1.563 mg/mL, which confirms the above results, although the classification of a lack of antimicrobial activity remains subject of debate.

Comparing the tested preparations, they were characterized by similar antimicrobial activity, despite a different tannin profiles. The data in Tables 6 and 7 clearly indicate that individual *L. monocytogenes* strains are characterized by different sensitivity to the preparations, and in extreme cases, the MIC and MBC values ​​between the selected strains differed 16- and 32-fold, respectively.

## Conclusions

The conducted studies have demonstrated that the extraction and purification technique applied to the Amberlite XAD 1600 bed yield highly concentrated polyphenolic preparations from raspberry and strawberry fruits, containing 74 g/100 g and 47 g/100 g of polyphenols, respectively, with a high proportion of tannins. The raspberry preparation (RTRP) is characterized by a high proportion of ellagitannins (88%), with the trimeric lambertianin C and the dimeric sanguiin H-6 being the dominant compounds. The strawberry preparation (STRP) is characterized by a similar proportion of proanthocyanidins and ellagitannins, at 53% and 43%, respectively, with the agrimoniin as the predominant compound. Our results confirm the antilisterial activity of these preparations, demonstrating their potential as natural antimicrobial agents. The effectiveness of these tannin-rich extracts suggests their applicability in food preservation strategies. Further optimization of extraction methods and formulation strategies will help enhance their efficiency and ensure their stability across different food systems.

## Supplementary Information

Supplementary Information 1.

## Data Availability

The research data were deposited in the open research data repository of Lodz University of Technology. Link to the repository: https://rdb.p.lodz.pl/dataverse/W5.

## References

[1] Farha, A. K. et al. Tannins as an alternative to antibiotics. *Food Bioscience*. **38**, 100751. 10.1016/j.fbio.2020.100751 (2020).

[2] Alexandri, M., Kachrimanidou, V., Papapostolou, H., Papadaki, A. & Kopsahelis, N. Sustainable food systems: The case of functional compounds towards the development of clean label food products. *Foods* 11, 2796. (2022). 10.3390/foods11182796 10.3390/foods11182796 PMC9498094 36140924

[3] Nowak, K., Jabłońska, E. & Ratajczak-Wrona, W. Controversy around parabens: alternative strategies for preservative use in cosmetics and personal care products. *Environ. Res.* **198**, 110488. 10.1016/j.envres.2020.110488 (2021). 33221305 10.1016/j.envres.2020.110488

[4] Hatano, T. et al. Effects of tannins and related polyphenols on methicillin-resistant *Staphylococcus aureus*. *Phytochemistry* **66**, 2047–2055. 10.1016/j.phytochem.2005.01.013 (2005). 16153408 10.1016/j.phytochem.2005.01.013

[5] Barbehenn, R. V. & Constabel, C. P. Tannins in plant-herbivore interactions. *Phytochemistry* **72**, 13, 1551–1565. 10.1016/j.phytochem.2011.01.040 (2011). 21354580 10.1016/j.phytochem.2011.01.040

[6] Quideau, S., Deffieux, D., Douat-Casassus, C. & Pouységu, L. Plant polyphenols: chemical properties, biological activities, and synthesis. *Angew. Chem. Int. Ed.* **50** (3), 586–621. 10.1002/anie.201000044 (2011). 10.1002/anie.201000044 21226137

[7] Gasperotti, M., Masuero, D., Vrhovsek, U., Guella, G. & Mattivi, F. Profiling and accurate quantification of rubus ellagitannins and ellagic acid conjugates using direct UPLC-Q-TOF HDMS and HPLC-DAD analysis. *J. Agric. Food Chem.* **58**, 4602–4616. 10.1021/jf904543w (2010). 20353173 10.1021/jf904543w

[8] Gasperotti, M. et al. Evolution of Ellagitannin content and profile during fruit ripening in *Fragaria* spp. *J. Agric. Food Chem.* **61**, 8597–8607. 10.1021/jf402706h (2013). 23992396 10.1021/jf402706h

[9] Kajdžanowska, M., Gjamovski, V. & Stefova, M. HPLC-DAD-ESI-MS^n^ identification of phenolic compounds in cultivated strawberries form Macedonia. *Maced. J. Chem. Chem. Eng.* **29** (2), 181–194 (2010).

[10] Gu, L. et al. Screening of foods containing proanthocyanidins and their structural characterization using LC-MS/MS and thiolytic degradation. *J. Agric. Food Chem.* **51**, 7513–7521. 10.1021/jf034815d (2003). 14640607 10.1021/jf034815d

[11] Puupponen-Pimiä, R. et al. Antimicrobial properties of phenolic compounds from berries. *J. Appl. Microbiol.* **90**, 494–507. 10.1046/j.1365-2672.2001.01271.x (2001). 11309059 10.1046/j.1365-2672.2001.01271.x

[12] Puupponen-Pimiä, R. et al. Berry phenolics selectively inhibit the growth of intestinal pathogens. *J. Appl. Microbiol.* **98**, 991–1000. 10.1111/j.1365-2672.2005.02547.x (2005). 15752346 10.1111/j.1365-2672.2005.02547.x

[13] Gullon, B., Pintado, M. E., Pérez-Álvarez, J. & Viuda-Martos, M. Assessment of polyphenolic profile and antibacterial activity of pomegranate Peel (*Punica granatum*) flour obtained from co-product of juice extraction. *Food Control*. **59**, 94–98. 10.1016/j.foodcont.2015.05.025 (2016).

[14] Xu, Y. et al. Tannin-rich pomegranate rind extracts reduce adhesion to and invasion of Caco-2 cells by *Listeria monocytogenes* and Decrease Its Expression of Virulence Genes. *J. Food. Prot.* **78** (1), 128–133. 10.4315/0362-028X.JFP-14-174 (2015). 25581187 10.4315/0362-028X.JFP-14-174

[15] Četojević-Simin, D. D. et al. Bioactivity of meeker and willamette raspberry (*Rubus idaeus* L.) pomace extracts. *Food Chem.* **166**, 407–413. 10.1016/j.foodchem.2014.06.063 (2015). 25053074 10.1016/j.foodchem.2014.06.063

[16] Marić, B. et al. UHPLC-Triple-TOF-MS characterization, antioxidant, antimicrobial and antiproliferative activity of raspberry (Rubus idaeus L.) seed extracts. *Foods* **12**, 161. 10.3390/foods12010161 (2023). 10.3390/foods12010161 PMC9818341 36613375

[17] Balgacem, I. et al. Effectiveness of a pomegranate peel extract (PGE) in reducing *Listeria monocytogenes* in vitro and on fresh–cut pear, apple and melon. *Eur. Food Res. Technol.* **246**, 1765–1772. 10.1007/s00217-020-03529-5 (2020).

[18] Klewicka, E. et al. Ellagitannins from raspberry (*Rubus idaeus* L.) fruit as natural inhibitors of *Geotrichum candidum*. *Molecules* **21** (7), 908. 10.3390/molecules21070908 (2016). 27420041 10.3390/molecules21070908 PMC6273995

[19] Sójka, M., Macierzyński, J., Zaweracz, W. & Buczek, M. Transfer and mass balance of ellagitannins, anthocyanins, Flavan-3-ols, and flavonols during the processing of red raspberries (*Rubus idaeus* L.) to Juice. *J. Agric. Food Chem.* **64**, 5549–5563. 10.1021/acs.jafc.6b01590 (2016). 27292440 10.1021/acs.jafc.6b01590

[20] Sójka, M., Klimczak, E., Macierzyński, J. & Kołodziejczyk, K. Nutrient and polyphenolic composition of industrial strawberry press cake. *Eur. Food Res. Technol.* **237**, 995–1007. 10.1007/s00217-013-2070-2 (2013).

[21] EUCAST Disk diffusion method for antimicrobial susceptibility testing version 12.0. - linkJanuary (2024).

[22] Naghili, H. et al. Validation of drop plate technique for bacterial enumeration by parametric and nonparametric tests. *Veterinary Res. Forum*. **4** (3), 179–183 (2013). PMC4312378 25653794

[23] Milczarek, A., Sójka, M. & Klewicki, R. Transfer of ellagitannins to unclarified juices and purees in the processing of selected fruits of the *Rosaceae* family. *Food Chem.* **344**, 128684. 10.1016/j.foodchem.2020.128684 (2021). 33272756 10.1016/j.foodchem.2020.128684

[24] Wani, T. A., Majid, D., Dar, B. M., Makroo, H. A. & Allai, F. M. Utilization of novel techniques in extraction of polyphenols from grape pomace and their therapeutic potential: a review. *J. Food Meas. Charact.* **17**, 5412–5425. 10.1007/s11694-023-02040-1 (2023).

[25] Milczarek, A., Sójka, M. & Klewicki, R. The kinetics of two–step ellagitannin extraction from the by–products of selected processed fruits of the family *Rosaceae*. *Food. Anal. Methods*. **15**, 1171–1184. 10.1007/s12161-021-02121-1 (2022).

[26] Rifna, E. J., Misra, N. N. & Dwivedi, M. Recent advances in extraction technologies for recovery of bioactive compounds derived from fruit and vegetable waste peels: A review. *Crit. Rev. Food Sci. Nutr.* **63** (6), 719–752. 10.1080/10408398.2021.1952923 (2023). 34309440 10.1080/10408398.2021.1952923

[27] Lee, G. E. et al. Optimization of accelerated solvent extraction of ellagitannins in black raspberry seeds using artificial neural network coupled with genetic algorithm. *Food Chem.* **396**, 133712. 10.1016/j.foodchem.2022.133712 (2022). 35863176 10.1016/j.foodchem.2022.133712

[28] Felix, A. C. S. et al. Alvarez L.D.G. An optimized alternative for phenolic compound-extraction of strawberry bagasse agro-industrial residues. *J. Microbiol. Biotechnol. Food Sci.* **8** (2), 815–820. 10.15414/jmbfs.2018.8.2.815-820 (2018).

[29] Kähkönen, M., Kylli, P., Ollilainen, V., Salminen, J-P. & Heinonen, M. Antioxidant activity of isolated ellagitannins from red raspberries and cloudberries. *J. Agric. Food Chem.* **60**, 1167–1174. 10.1021/jf203431g (2012). 22229937 10.1021/jf203431g

[30] Fotschki, B. et al. Strawberry ellagitannins thwarted the positive effects of dietary fructooligosaccharides in rat Cecum. *J. Agric. Food Chem.* **62**, 5871–5880. 10.1021/jf405612a (2014). 24894695 10.1021/jf405612a

[31] Salazar-Orbea, G. L. et al. Stability of phenolic compounds in apple and strawberry: Effect of different processing techniques in industrial set up. *Food Chem.* **401**, 134099. 10.1016/j.foodchem.2022.134099 (2023). 36099818 10.1016/j.foodchem.2022.134099

[32] Barbera, G. L. et al. Comprehensive polyphenol profiling of a strawberry extract (*Fragaria × ananassa*) by ultra-high-performance liquid chromatography coupled with high-resolution mass spectrometry. *Anal. Bioanal. Chem.* **409**, 2127–2142. 10.1007/s00216-016-0159-8 (2017). 28078420 10.1007/s00216-016-0159-8

[33] Jing, W., Xiaolan, C., Yu, C., Feng, Q. & Haifeng, Y. Pharmacological effects and mechanisms of tannic acid. *Biomed. Pharmacother.* **154**, 113561. 10.1016/j.biopha.2022.113561 (2022). 36029537 10.1016/j.biopha.2022.113561

[34] Wu, D., Wu, X. D., You, X. F., Ma, X. F. & Tian, W. X. Inhibitory effects on bacterial growth and beta-ketoacyl-ACP reductase by different species of maple leaf extracts and tannic acid. *Phytother. Res.* **24** (Suppl. 1), S35–41. 10.1002/ptr.2873 (2010). 19444866 10.1002/ptr.2873

[35] Oliveira, B. D. Á. et al. Antioxidant, antimicrobial and anti-quorum sensing activities of *Rubus rosaefolius* phenolic extract. *Ind. Crops Prod.* **84**, 59–66. 10.1016/j.indcrop.2016.01.037 (2016).

[36] Ispiryan, A. et al. Correlation between antimicrobial activity values and total phenolic content/antioxidant activity in *Rubus idaeus* L. *Plants* 13, 504. (2024). 10.3390/plants13040504 10.3390/plants13040504 PMC10891700 38498473

[37] Khalifa, H. O., Kamimoto, M., Shimamoto, T. & Shimamoto, T. Antimicrobial effects of blueberry, raspberry, and strawberry aqueous extracts and their effects on virulence gene expression in *Vibrio cholerae*. *Phytother. Res.* **29**, 1791–1797. 10.1002/ptr.5436 (2015). 26292998 10.1002/ptr.5436

[38] Barbieri, F. et al. Effects of Rubus fruticosus and Juniperus oxycedrus derivatives on culturability and viability of Listeria monocytogenes. *Sci. Rep.* **12** (13158). 10.1038/s41598-022-17408-4 (2022). 10.1038/s41598-022-17408-4 PMC9343658 35915316

[39] Shimozu, Y. et al. Ellagitannins of *Davidia involucrata*. I. Structure of davicratinic acid A and effects of davidia tannins on drug-resistant bacteria and human oral squamous cell carcinomas. *Molecules* **22**, 420. 10.3390/molecules22030470 (2017). 28294988 10.3390/molecules22030470 PMC6155176

[40] Li, G. et al. Tannin-rich fraction from pomegranate rind damages membrane of *Listeria monocytogenes*. *Foodborne Pathog. Dis.* **11** (4), 1–7. 10.1089/fpd.2013.1675 (2014). 24447173 10.1089/fpd.2013.1675

[41] Funatogawa, K. et al. Antibacterial activity of hydrolyzable tannins derived from medicinal plants against *Helicobacter pylori*. *Microbiol. Immunol.* **48** (4), 251–261. 10.1111/j.1348-0421.2004.tb03521.x (2004). 15107535 10.1111/j.1348-0421.2004.tb03521.x

[42] Savic, I. M. et al. The effect of complexation with cyclodextrins on the antioxidant and antimicrobial activity of ellagic acid. *Pharm. Dev. Technol.* **24** (4), 410–418. 10.1080/10837450.2018.1502318 (2019). 30035651 10.1080/10837450.2018.1502318

[43] Caballero, V. et al. Biodegradation of punicalagin into allagic acid by selected probiotic bacteria: A study of the underlying mechanisms by MS-based proteomics. *J. Agric. Food Chem.* **70**, 16273–16285. 10.1021/acs.jafc.2c06585 (2022). 36519204 10.1021/acs.jafc.2c06585 PMC9801417

[44] Aguilera-Carbo, A., Augur, C., Prado-Barragan, L. A., Favela-Torres, E. & Aguilar, C. N. Microbial production of ellagic acid and biodegradation of ellagitannins. *Appl. Microbiol. Biotechnol.* **78**, 189–199. 10.1007/s00253-007-1276-2 (2008). 18157721 10.1007/s00253-007-1276-2

[45] Rappin, S. A. K. & Beniwal, V. Reconnoitring the antioxidant and antibacterial potential of different fruits after tannin acyl hydrolase mediated biotransformation. *Biotechnol. Appl. Chem.* **70**, 1436–1449. 10.1002/bab.2461 (2023). 10.1002/bab.2461 36965069

[46] Alejo-Armijo, A. et al. Antimicrobial and antibiofilm activities of procyanidins extracted from laurel wood against a selection of foodborne microorganisms. *Int. J. Food Sci. Technol.* **52**, 679–686. 10.1111/ijfs.13321 (2017).

[47] Wang, C-M. et al. Structure elucidation of procyanidins isolated from rhododendron formosanum and their anti-oxidative and anti-bacterial activities. *Molecules* **20** (7), 12787–12803. 10.3390/molecules200712787 (2015). 26184152 10.3390/molecules200712787 PMC6332352

[48] Dakheel, M. M., Alkandari, F. A. H., Mueller-Harvey, I., Woodward, M. J. & Rymer, C. Antimicrobial in vitro activities of condensed tannin extracts on avian pathogenic *Escherichia coli*. *Lett. Appl. Microbiol.* **70** (3), 165–172. 10.1111/lam.13253 (2020). 31782190 10.1111/lam.13253

[49] Sivakumaran, S. et al. Variation in antimicrobial action of proanthocyanidins from *Dorycnium rectum* against rumen bacteria. *Phytochemistry* **65**, 2485–2497. 10.1016/j.phytochem.2004.08.046 (2004). 15381413 10.1016/j.phytochem.2004.08.046

[50] Koll, M. M., Comeskey, D. J., Cooney, J. M. & McGhie, T. K. Structural identification of the main ellagitannins of a boysenberry (*Rubus loganbaccus* × *baileyanus* Britt.) extract by LC–ESI-MS/MS, MALDI-TOF-MS and NMR spectroscopy. *Food Chem.* **119**, 1535–1543. 10.1016/j.foodchem.2009.09.039 (2010).

[51] Kula, M. & Krauze-Baranowska, G. D. Two-dimensional liquid chromatography (LC) of phenolic compounds from the shoots of *Rubus idaeus* ‘Glen Ample’ cultivar variety. *J. Pharm. Biomed. Anal.* **121**, 99–106. 10.1016/j.jpba.2015.12.047 (2016). 26799975 10.1016/j.jpba.2015.12.047

[52] Hager, T. J., Howard, L. R., Liyanage, R., Lay, J. O. & Prior, R. L. Ellagitannin composition of blackberry as determined by HPLC-ESI-MS and MALDI-TOF-MS. *J. Agric. Food Chem.* **56**, 661–669. 10.1021/jf071990b (2008). 18211030 10.1021/jf071990b

[53] McDougall, G. J. et al. Gill C.I.R. Tracking (poly)phenol components from raspberries in ileal fluid. *J. Agric. Food Chem.* **62**, 7631–7641. 10.1021/jf502259j (2014). 24998385 10.1021/jf502259j

[54] Arapitsas, P., Menichetti, S., Vincieri, F. F. & Romani, A. Hydrolyzable tannins with the hexahydroxydiphenoyl unit and the m-depsidic Link: HPLC-DAD-MS identification and model synthesis. *J. Agric. Food Chem.* **55**, 48–55. 10.1021/jf0622329 (2007). 17199312 10.1021/jf0622329

[55] Karlińska, E., Pecio, Ł., Macierzyński, J., Stochmal, A. & Kosmala, M. Structural elucidation of the ellagitannin with a molecular weight of 2038 isolated from strawberry fruit (*Fragaria ananassa* Duch.) and named fragariin A. *Food Chem.* **296**, 109–115. 10.1016/j.foodchem.2019.05.191 (2019). 31202294 10.1016/j.foodchem.2019.05.191

[56] Chen, Y. et al. Identification of ellagitannins in the unripe fruit of *Rubus Chingii* Hu and evaluation of its potential antidiabetic activity. *J. Agric. Food Chem.* **67**, 7025–7039. 10.1021/acs.jafc.9b02293 (2019). 31240933 10.1021/acs.jafc.9b02293

[57] Macierzyński, K., Sójka, M., Kosmala, M. & Karlińska, E. Transformation of oligomeric ellagitannins, yypical for rubus and fragaria genus, during strong acid hydrolysis. *J. Agric. Food Chem.* **68**, 8212–8222. 10.1021/acs.jafc.0c02674 (2020). 32648752 10.1021/acs.jafc.0c02674 PMC7458417

[58] Enomoto, H. Unique distribution of ellagitannins in ripe strawberry fruit revealed by mass spectrometry imaging. *Curr. Res. Food Sci.* **4**, 821–828. 10.1016/j.crfs.2021.11.006 (2021). 34841268 10.1016/j.crfs.2021.11.006 PMC8606305

[59] Kårlund, A. et al. Polyphenols in strawberry (*Fragaria × ananassa*) leaves induced by plant activators. *J. Agric. Food Chem.* **62**, 4592–4600. 10.1021/jf405589f (2014). 24819677 10.1021/jf405589f

[60] Nowicka, A., Kucharska, A. Z., Sokół-Łetowska, A. & Fecka, I. Comparision of polyphenol content and antioxidant capacity of strawberry fruit from 90 cultivars of *Fragaria x ananasa* Duch. *Food Chem.* **270**, 32–46. 10.1016/j.foodchem.2018.07.015 (2019). 30174053 10.1016/j.foodchem.2018.07.015

[61] Abby, K., Mazur, S., Nes, A. & Skrede, G. Phenolic compounds in strawberry (*Fragaria x ananassa* Duch.) fruits: Composition in 27 cultivars and changes during ripening. *Food Chem.* **132**, 86–97. 10.1016/j.foodchem.2011.10.037 (2012). 26434267 10.1016/j.foodchem.2011.10.037

[62] Duckstein, S. M., Lotter, E. M., Meyer, U. & Lindequist, Stintzing, F. C. Phenolics constituents Alchemilla vulgaris L. and Alchemilla mollis (Buser) Rothm. at different dates of harvest. *Z. Naturforsch* 67 c, 529–540. (2012). 23413745

[63] Chen, L., Xin, X., Zhang, H. & Yuan, Q. Phytochemical properties and antioxidant capacities of commercial raspberry varieties. *J. Funct. Foods.* **5**, 508–515. 10.1016/j.jff.2012.10.009 (2013).

[64] Mullen, W., Larcombe, S., Arnold, K., Welchman, H. & Crozier, A. Use of accurate mass full scan mass spectrometry for the analysis of anthocyanins in berries and berry-fed tissues. *J. Agric. Food Chem.* **58**, 3910–3915. 10.1021/jf902267v (2010). 20014766 10.1021/jf902267v

[65] Mustafa, A. M. et al. A new HPLC-MS/MS method for the simultaneous determination of 36 polyphenols in blueberry, strawberry and their commercial products and determination of antioxidant activity. *Food Chem.* **367**, 130743. 10.1016/j.foodchem.2021.130743 (2022). 34384982 10.1016/j.foodchem.2021.130743

