# Supplementary figures and images for: Antilisterial activity of tannin rich preparations isolated from raspberry (Rubus Idaeus L.) and strawberry (Fragaria X Ananassa Duch.) fruit
Source: Sci Rep. 2025 Mar 25;15:10196. doi: 10.1038/s41598-025-94731-6 (PMC11937420; doi:10.1038/s41598-025-94731-6)

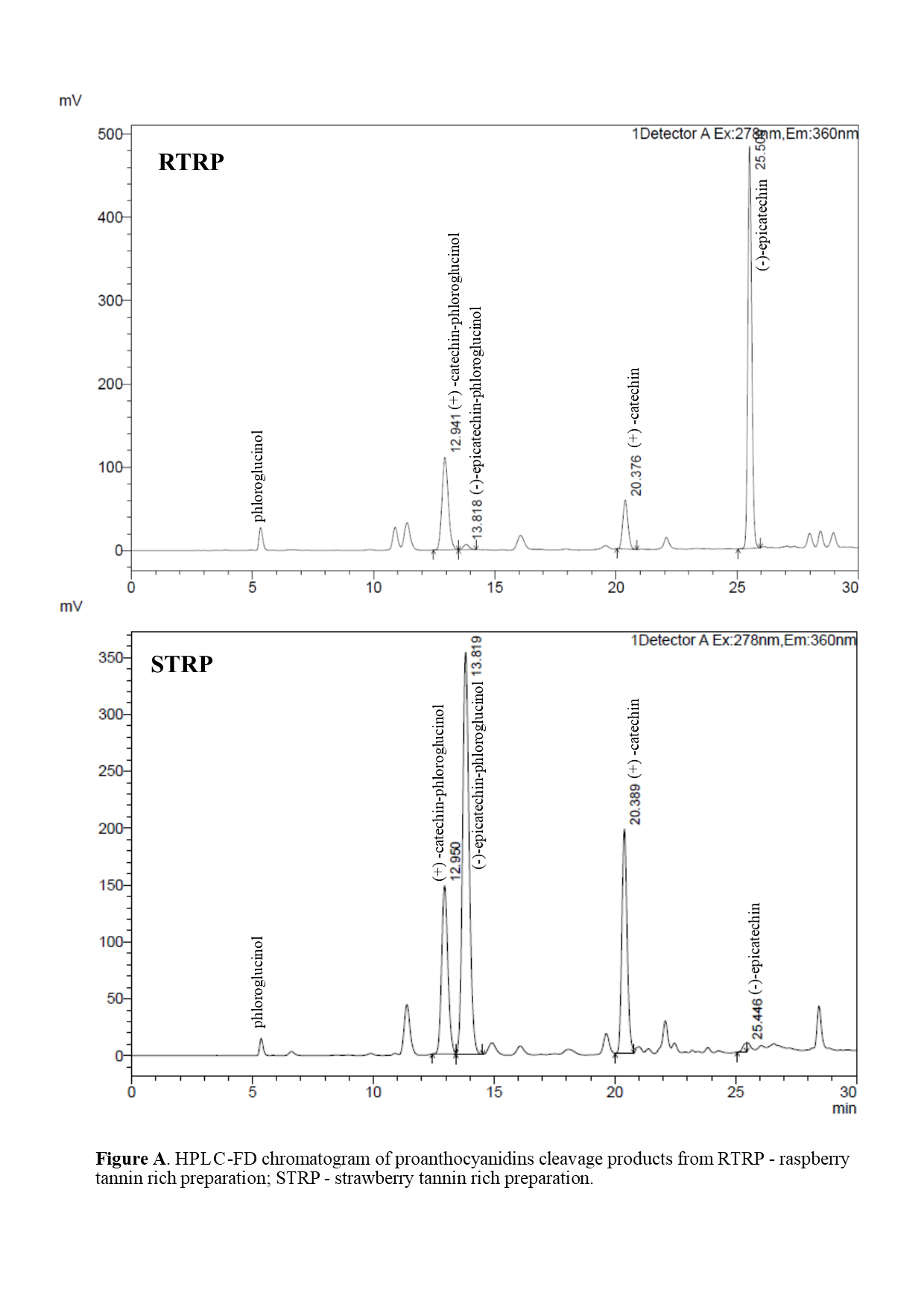

Supplement: Supplementary file 1 — Supplementary Information 1. [file 41598_2025_94731_MOESM1_ESM.png]
